# Establishment of *pten* knockout medaka with transcription activator–like effector nucleases (TALENs) as a model of PTEN deficiency disease

**DOI:** 10.1371/journal.pone.0186878

**Published:** 2017-10-20

**Authors:** Yuriko Matsuzaki, Tetsushi Sakuma, Takashi Yamamoto, Hideyuki Saya

**Affiliations:** 1 Division of Gene Regulation, Institute for Advanced Medical Research, School of Medicine, Keio University, Tokyo, Japan; 2 Department of Mathematical and Life Sciences, Graduate School of Science, Hiroshima University, Hiroshima, Japan; Texas A&M University, UNITED STATES

## Abstract

Phosphatase and tensin homolog (PTEN) is a lipid and protein phosphatase that antagonizes signaling by the phosphatidylinositol 3-kinase (PI3K)–AKT signaling pathway. The *PTEN* gene is a major tumor suppressor, with mutations of this gene occurring frequently in tumors of humans and mice. We have now developed mutant medaka deficient in PTEN with the use of transcription activator–like effector nuclease (TALEN) technology. Medaka possesses two *pten* genes, *ptena* and *ptenb*, similar to zebrafish. We established 16 *ptena* mutant lines and two *ptenb* mutant lines. Homozygous single *pten* mutants were found to be viable and fertile. In contrast, *pten* double-knockout (dko) embryos manifested severe abnormalities in vasculogenesis, eye size, and tail development at 72 hours post fertilization(hpf) and died before hatching. Immunoblot analysis revealed that the ratio of phosphorylated to total forms of AKT (pAKT/AKT) in *pten* dko embryos was four times that in wild-type embryos, indicative of up-regulation of signaling by the PI3K-AKT pathway. Treatment of *pten* dko embryos with the PI3K inhibitor LY294002 reduced the pAKT/AKT ratio by about one-half and partially rescued the defect in vasculogenesis. Additional inhibitors of the PI3K-AKT pathway, including rapamycin and *N*-α-tosyl-L-phenylalanyl chloromethyl ketone, also partially restored vasculogenesis in the dko embryos. Our model system thus allows *pten* dko embryos to be readily distinguished from wild-type embryos at an early stage of development and is suitable for the screening of drugs able to compensate for PTEN deficiency.

## Introduction

Phosphatase and tensin homolog (PTEN) is a lipid and protein phosphatase that catalyzes the dephosphorylation of phosphatidylinositol 3,4,5-trisphosphate and thereby antagonizes signaling by the phosphatidylinositol 3-kinase (PI3K)–AKT signaling pathway[1–3]. The *PTEN* gene is a major tumor suppressor, with mutations of this gene having been frequently detected in tumors of humans and mice. Germline mutations in *PTEN* give rise to PTEN hamartoma tumor syndrome (PHTS), which includes Cowden syndrome, Bannayan-Riley-Ruvalcaba syndrome, Proteus syndrome, and Proteus-like syndrome[4,5]. The development of molecularly targeted drugs for conditions caused by mutant forms of PTEN is a priority, with such targeted agents being first identified by screening at the cell or molecular level and then tested in animals, usually mice, before entry into clinical trials. We have previously developed a screening system based on *HRAS* transgenic medaka for the testing of anticancer drugs [6]. Zebrafish is often adopted as a vertebrate model for chemical or genetic screening because it is readily raised in large numbers and its embryos are transparent [7–9]. Medaka possesses these same characteristics and has also been used as a vertebrate model of human disease [10,11].

Zebrafish possesses two PTEN genes, *ptena* and *ptenb*, with double-homozygous *pten* mutants having been shown to die at 5 days post fertilization(dpf) manifesting pleiotropic defects and enhanced cell survival [12]. Furthermore, angiogenesis and expression of the *vascular endothelial growth factor (VEGF)* gene were also enhanced in *ptena*^−/−^*ptenb*^−/−^ mutant embryos, and this hypervascularization phenotype was ameliorated by combined treatment with the VEGF receptor inhibitor sunitinib and the PI3K inhibitor LY294002 [13]. The hypervascularization phenotype was also applied to differentiate the role of the lipid and protein phosphatase activity of PTEN during embryonic development [14]. Database sequences suggest that medaka also possesses two *PTEN* genes (*ptena* and *ptenb*), with *ptena* being located on chromosome 8 but the chromosomal location of *ptenb* being unknown.

Transcription activator–like effector nucleases (TALENs) were developed as the genome editing tool, comprising *Xanthomonas* transcription activator–like effector (TALE) protein and *Fok*I nuclease domain [15]. The advantage of TALEN technology is its high specificity of sequence recognition compared with the CRISPR/Cas9 system [16]. TALEN can typically recognize 15- to 20-bp DNA sequence for the left and right monomer each; thus, a total of 30- to 40-bp sequence is specifically recognized, which accounts for the minimal off-target effects of this technology. We have now applied TALEN technology to generate *pten* knockout medaka with target sequences localized to the phosphatase domain of *ptena* and *ptenb*. We obtained *pten* knockout medaka with high efficiency, and we found that the double-knockout (*ptena*^−/−^*ptenb*^−/−^; dko) progeny derived from a cross between the single-knockout strains manifested severe abnormalities that are likely to prove applicable to drug screening.

## Materials and methods

### Ethics statement

All animal experiments were performed in accordance with protocols approved by the Animal Care and Use Committee of Keio University. This study was approved as permit no. 12038-4 by the Animal Care and Use Committee of Keio University.

### Medaka maintenance

Medaka (*Oryzias latipes*) strain OK-Cab (strain ID, MT830) was obtained from NBRP Medaka (https://shigen.nig.ac.jp/medaka) and was maintained according to established protocols (https://shigen.nig.ac.jp/medaka/medakabook). Fish were anaesthetized with 0.02% tricaine methanesulfonate, and were sacrificed with 0.2% tricaine methanesulfonate.

### Extraction of total RNA and RT-PCR analysis

Total RNA was isolated from medaka embryos with the use of an RNeasy Mini Kit (Qiagen, Hilden, Germany) and an RNase-Free DNase Set (Qiagen) in order to eliminate genomic DNA from the samples. The extracted RNA was subjected to RT with the use of a Transcriptor First Strand cDNA Synthesis Kit (Roche Applied Science, Rotkreuz, Switzerland), and the presence of medaka *pten* cDNAs among the RT products was determined with a medaka *ptena* primer set (*ptena*-S, 5'-ATGGTCAGTCGGAACAAAAGGAGATAC-3'; *ptena*-E, 5'-CACTTTTGTGATTTGCGTGTGCTGCTC-3') and a medaka *ptenb* primer set (*ptenb*-S, 5'-ATGGCCGCCAATCTAATCAAGGAGATCGTG-3'; *ptenb*-E, 5'-ATCTGTTCGTGGTCGTCCTCCTC-3'). The amplification protocol comprised an initial incubation at 94°C for 1 min; 35 cycles of 98°C for 10 s, 56°C for 20 s, and 72°C for 75 s; and a final incubation at 72°C for 7 min. The reaction mixture consisted of 2 μl of 10×LA buffer (Takara, Kyoto, Japan), 0.5 μl of cDNA, 0.5 μM of each primer, and 0.5 U of LA Taq DNA Polymerase (Takara) in a total volume of 20 μl.

### Plasmid construction and in vitro transcription

Gene disruption was performed with the TALEN system. TALEN plasmids were constructed using the Platinum Gate TALEN Kit (Addgene, Cambridge, MA, USA; Kit #1000000043) as described previously[17] with modifications described as follows: the plasmid backbone of ptCMV-136/63-VR vectors were replaced with pCS2P, containing SP6 promoter for in vitro transcription. Medaka *ptena* and *ptenb* TALEN constructs were digested with *Not*I (Thermo Fisher Scientific, Waltham, MA, USA) and were transcribed in vitro with the use of a mMessage mMachine SP6 transcription kit (Thermo Fisher Scientific).

### Microinjection of mRNA

Fertilized eggs from OK-Cab adult fish were injected with ~5 nl of a mixture containing 40 to 125 pg of mRNA, 0.5× Yamamoto’s medium, and 0.05% phenol red. Embryos were raised and outcrossed with wild-type fish to identify animals with mutations.

### Extraction of genomic DNA

Genomic DNA was extracted from the fin of adult fish or from the entire body of larvae. The tissue samples were incubated for 10 min at room temperature in 100 μl of a lysis buffer containing 20 mM Tris-HCl (pH 8.0), 5 mM EDTA, 400 mM NaCl, 0.3% SDS, and proteinase K (Nacalai tesque, Kyoto, Japan), after which the lysates were heated for 5 min at 95°C and subjected directly to PCR.

### Determination of an effective mRNA concentration for microinjection, and selection of *ptena* and *ptenb* mutant medaka

The *pten* target regions of G0 individuals were amplified and then cloned into a T-overhangs vector (pGEM-T Easy; Promega, Madison, WI, USA). The sequences of inserted fragments were determined for four individual clones from each of five embryos injected at each mRNA concentration. The *pten* mutations of F1 individuals were checked by PCR analysis of the targeted regions and digestion with restriction enzymes. The primer sequences for the targeted regions are as follows: *ptena*-forward, 5'-ATGCCGTCACGCATCAGTTGCA-3'; *ptena*-reverse, 5'-ACAGACGTTTGAACCCACCTTC-3'; *ptenb*-forward, 5'-CTGCGTCTCTTTCAGTGGCCC-3'; and *ptenb*-reverse, 5'-CACGAGCCACCCTCACCTTCTT-3'. The amplification protocol comprised an initial incubation at 94°C for 2 min; 35 cycles of 98°C for 10 s, 58.5°C for 20 s, and 68°C for 30 s; and a final incubation at 68°C for 7 min. The reaction mixture consisted of 10 μl of 2× PCR buffer (Toyobo, Osaka, Japan), 0.5 μl of genomic DNA, 0.4 μM of each primer, and 0.4 U of KOD FX Neo (Toyobo) in a total volume of 20 μl. The PCR product of *ptena* was digested with *Pvu*II and *Fok*I (Thermo Fisher Scientific), and that of *ptenb* was digested with *Pvu*II and *Hpy*F3I (Thermo Fisher Scientific).

### Histological and immunohistochemical staining

The entire fish body was fixed in Davidson’s solution (31% ethanol, 8% formaldehyde, 11.5% acetic acid), embedded in paraffin, serially sectioned, and stained with hematoxylin-eosin for histological analysis. For immunohistochemistry, serial sections with a thickness of 4 μm were stained with the use of a Vectastain ABC Elite kit (Vector Laboratories, Burlingame, CA, USA). The sections were thus stained with antibodies to pAKT (#4060; Cell Signaling Technology, Danvers, MA, USA), to pGSK-3β (#9323, Cell Signaling Technology), to CD31 (Ab28364; Abcam, Cambridge, UK), and to Proliferating cell nuclear antigen (NCL-L-PCNA, Leica Biosystems, Newcastle, UK), and they were counterstained with hematoxylin to visualize cell nuclei. Images were captured with the use of a Biorevo BZ-9000 microscope (Keyence, Osaka, Japan).

### Immunoblot analysis

Protein samples extracted from dorsal muscle of adult fish at 7 to 10 mpf or from whole embryos were subjected to SDS-polyacrylamide gel electrophoresis, and the separated proteins were transferred to a polyvinylidene difluoride membrane (Immobilon-P; Millipore, Bedford, MA, USA). The membrane was incubated overnight at 4°C with primary antibodies including those to pAKT (1:1000 dilution; #4060, Cell Signaling Technology), to AKT (1:1000 dilution; #9272, Cell Signaling Technology), to β-actin (1:500 dilution, C4, sc-47778; Santa Cruz Biotechnology, Dallas, TX, USA), or to α-tubulin (1:500 dilution, DM1A, sc-32293; Santa Cruz Biotechnology). Incubation with horseradish peroxidase–conjugated secondary antibodies was performed for 1 h at room temperature. Peroxidase activity was detected with chemiluminescence reagents (Chemi-Lumi One, Nacalai tesque). The intensity of immunoreactive bands was measured with the use of an LAS-3000 Mini instrument and Multi Gauge version 3.0 software (Fujifilm, Tokyo, Japan).

### Drug treatment

Before drug exposure, fertilized eggs were rubbed with waterproof sandpaper for partial dechorionation. They were then treated in multiwell plates and at the indicated times with 15 μM LY294002 (LC Laboratories, Woburn, MA, USA), 5 μM rapamycin (Toronto Research Chemicals, Toronto, Ontario, Canada) or 2 μM TPCK (Wako, Osaka, Japan) in fish water. In preliminary experiments, we found that treatment of medaka embryos with 30 μM LY294002, 10 μM rapamycin, or 5 μM TPCK for 48 h was lethal or caused developmental retardation.

### Photography

Fish were photographed with a Leica DFC300FX camera, and images were processed with Leica Application Suite version 2.7.1.R1 software (Leica Microsystems, Wetzlar, Germany). Moving images were captured with a Biorevo BZ-9000 microscope (Keyence).

### Statistical analysis

The unit of analysis for each dataset is single animal. Statistical analysis was performed with Student’s *t* test. A *P* value of <0.05 was considered statistically significant.

## Results

### Design of TALEN constructs for the two PTEN genes of medaka

**Fig 1:**
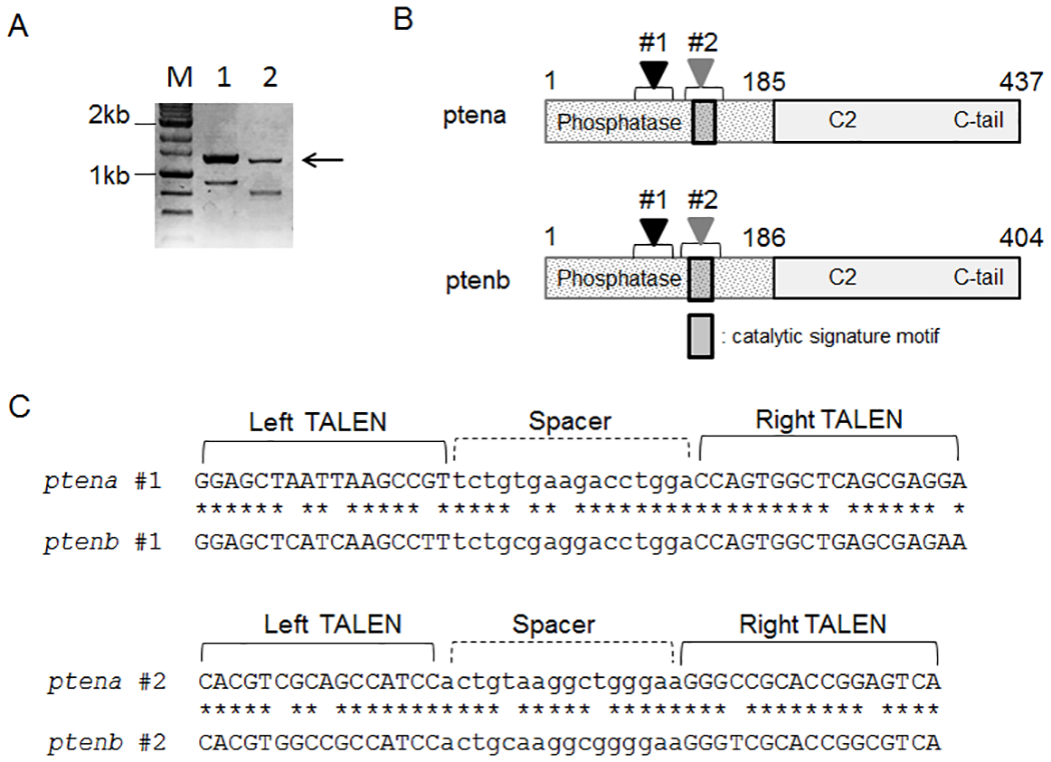
Characterization of medaka PTEN genes and design of TALEN constructs. **(A**) RT-PCR analysis of total RNA isolated from whole embryos of medaka was performed with two sets of primers designed to amplify almost the entire open reading frames of *ptena* (lane 1) and *ptenb* (lane 2). The arrow indicates the specific amplification products, with the faster-migrating bands being found to represent technical artifacts by sequencing analysis. Lane M, molecular size markers. (**B**) Products of medaka *ptena* and *ptenb* predicted from the sequences of the amplified cDNAs and database information. Arrowheads labeled #1 and #2 indicate the localization of the TALEN target sequences shown in (C). (**C**) TALEN target sites in *ptena* and *ptenb*.

The existence of two PTEN genes in medaka is predicted from sequences in the medaka genome database (LOC101171856 and LOC101173689). The similarity of these sequences to *ptena* and *ptenb* of zebrafish indicates that LOC101171856 corresponds to *ptena* and LOC101173689 to *ptenb* of medaka. We verified the production of transcripts from these two putative *pten* genes by reverse transcription (RT) and polymerase chain reaction (PCR) analysis performed with total RNA isolated from whole embryos of wild-type fish and with two sets of primers designed to amplify almost the entire open reading frames of *ptena* (1290-bp product) and *ptenb* (1262-bp product) (Fig 1A). The products of *ptena* and *ptenb* were predicted from the sequences of the amplified cDNAs and database sequences (Fig 1B). The predicted amino acid sequences of medaka Ptena and Ptenb are 81.5% and 81.1% identical, respectively, to that of human PTEN. The medaka Ptena and Ptenb proteins also share 85.7% and 76.2% identity with the corresponding zebrafish proteins. We designed four TALEN sets to introduce deletions at two positions within the phosphatase domain of medaka *ptena* and *ptenb* genes (Fig 1C). Regarding the TALENs targeting the position #1, three and two mismatches exist in the left and right target sequences, respectively, between *ptena* and *ptenb* genes. Regarding the TALENs #2, two mismatches exist both in the left and right target sequences. Thus, *ptena* #1 and *ptena* #2 TALENs might be specific for the *ptena* gene, while *ptenb* #1 and *ptenb* #2 TALENs might be specific for the *ptenb* gene.

### Establishment of *pten* knockout medaka with TALEN technology

**Fig 2:**
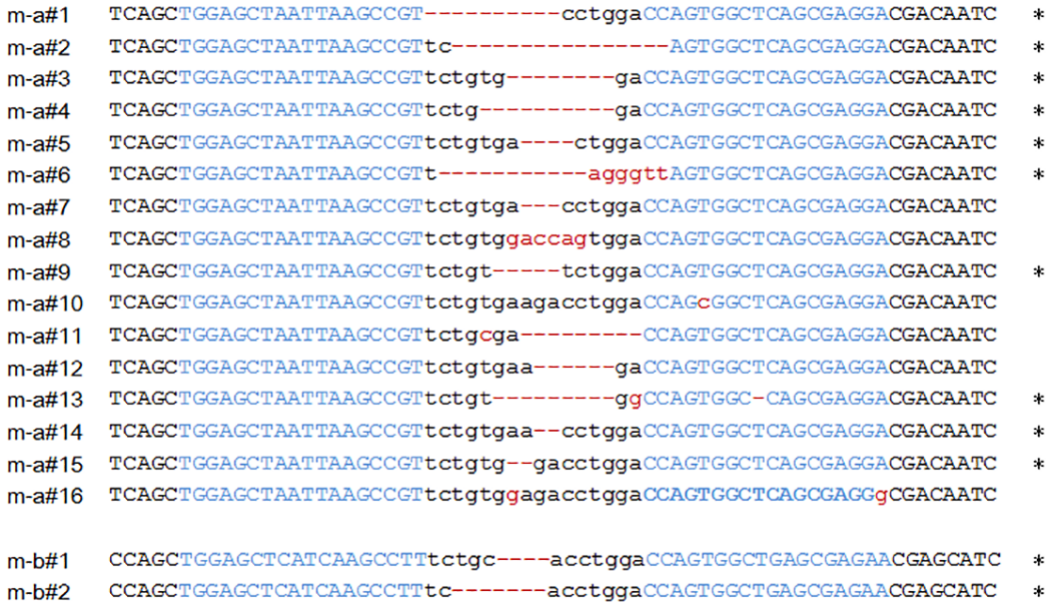
Genomic DNA sequences of the TALEN target sites in F1 *ptena* (m-a#) or *ptenb* (m-b#) mutant medaka clones. Most clones had deletions (dashes), and some had nucleotide changes (red lowercase letters). Asterisks indicate clones with out-of-frame deletions.

The mRNAs synthesized by in vitro transcription of the *pten* TALEN constructs were introduced at concentrations of 8 to 25 μg ml^–1^ into medaka embryos at the one-cell stage. Genomic sequencing of G0 embryos revealed 15 mutations at the *ptena* locus derived from five fish with the *ptena* #1 construct at 25 μg ml^–1^ and five mutations at the *ptenb* locus derived from four fish with the *ptenb* #1 construct at 10 μg ml^–1^ (Table 1). Neither the *ptena* #2 nor *ptenb* #2 construct efficiently introduced mutations into the medaka genome. Furthermore, given that injection of mRNAs at 25 μg ml^–1^ resulted in failure of 80% of embryos to hatch, we decided to use *ptena* #1 and *ptenb* #1 constructs at 10 μg ml^–1^ for future experiments. The modified G0 embryos were raised to adult fish, and the resulting mature animals were outcrossed with wild-type fish for mutational analysis. We established 18 medaka strains with *ptena* or *ptenb* mutations at the TALEN target sites (Fig 2). Most of the mutations were localized in the spacer region, although some were detected in the right arm region. Ten *ptena* strains and two *ptenb* strains had out-of-frame deletions. Some *ptena* or *ptenb* heterozygous or homozygous adult fish manifested muscle hyperplasia, abnormal osteogenesis, or a thin body (S1 Fig), although the frequency of these phenotypes was low (<10%) and their onset late (~7 months post-fertilization (mpf) or later).

**Table 1:** Mutation efficiency for *pten* TALEN constructs revealed by analysis of G0 medaka embryos.

TALEN construct	Concentration of mRNA (μg ml^–1^)	No. of mutated embryos/5	No. of mutations
***ptena* #1**	25	5	15
	12.5	4	6
	8	4	12
***ptena* #2**	25	1	1
***ptenb* #1**	10	4	5
***ptenb* #2**	10	0	0

### Generation of *pten* double-knockout medaka

We crossed a *ptena* mutant with an 8-nucleotide deletion (m-a#3) and a *ptenb* mutant with a 4-nucleotide deletion (m-b#1) to generate a *pten* double-knockout (dko) medaka (Fig 2). Both mutant alleles encoded PTEN proteins consisting of a truncated phosphatase domain and are hereafter designated *ptena*^−^ and *ptenb*^−^, respectively. The cross was performed with *ptena*^−/−^*ptenb*^+/−^ and *ptena*^+/−^*ptenb*^−/−^ fish. The resultant F1 fish were raised to young (2 mpf) or adult fish (3 mpf), and their genotype was confirmed (S1 Table). Whereas the theoretical segregation ratio of F1 *ptena*^−/−^*ptenb*^−/−^ fish is 0.25, we did not detect any individuals of this genotype among the 95 fish analyzed. The observed segregation ratio for each mutation (*ptena*^−^ and *ptenb*^−^) was also significantly different from the theoretical ratio. Given that *ptena*^−/−^*ptenb*^+/−^ individuals had a low viability and were difficult to maintain, we subsequently crossed *ptena*^+/−^*ptenb*^−/−^ fish with each other in order to obtain *pten* dko mutants.

### PI3K-AKT signaling in adult *pten* mutant fish

**Fig 3:**
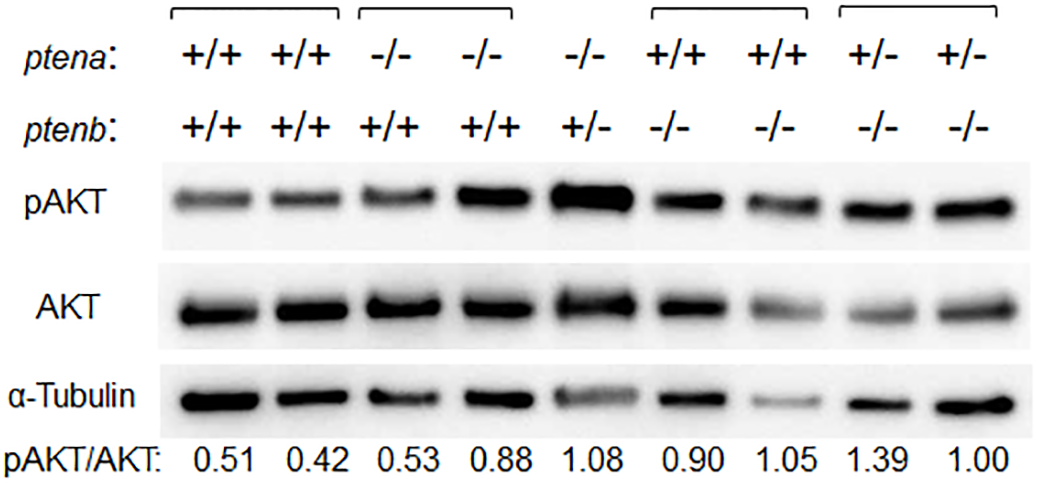
PI3K-AKT signaling pathway activity in adult *pten* mutant medaka. Extracts (20 μg of protein) derived from the dorsal muscle of 7- to 10-mpf fish were subjected to immunoblot analysis with antibodies to phosphorylated (p) or total forms of AKT as well as with those to α-tubulin (loading control). Each lane corresponds to an individual. The pAKT/AKT ratio for individual fish of the indicated *pten* genotypes was determined by densitometry. There were another two independent replications that showed much the same results.

Protein extracts prepared from the dorsal muscle of adult fish were subjected to immunoblot analysis of total and phosphorylated (activated) forms of AKT (Fig 3). Given that the phosphorylation of AKT is inhibited by PTEN, the ratio of phosphorylated AKT to total AKT (pAKT/AKT) reflects PTEN function. The pAKT/AKT ratio was increased in *ptena*^−/−^*ptenb*^+/−^ (1.08), *ptena*^+/−^*ptenb*^−/−^ (1.39, 1.00), and *ptena*^+/+^*ptenb*^−/−^ (0.90, 1.05) medaka compared with wild-type fish (0.51, 0.42), whereas that in *ptena*^−/−^*ptenb*^+/+^ fish (0.53, 0.88) was similar to that of the wild type. Similar results were obtained in three repeated experiments. If the pAKT/AKT ratio for the wild type is set to 1.0, the average value of the ratio determined from the three experiments was 1.97 for *ptena*^−/−^*ptenb*^+/−^ fish and 1.84 for *ptena*^+/−^*ptenb*^−/−^ individuals.

### Phenotypes and histology of *ptena*^+/–^*ptenb*^−/−^ adult fish

A small proportion of *ptena*^+/−^*ptenb*^−/−^ fish developed tumors (3/50, 6%) or manifested abnormal osteogenesis (6/50, 12%) at 4 to 8 mpf (S2A–S2D Fig). Serial sections of tumor sites were examined by hematoxylin-eosin staining and immunohistochemical analysis of pAKT, phosphorylated glycogen synthase kinase–3β (pGSK-3β, with phosphorylation of GSK-3β being mediated by AKT in the PI3K-AKT pathway), CD31 (a marker of vascular endothelial cells), and proliferating cell nuclear antigen (PCNA) (S2E–S2N Fig). The marginal region of tumor tissue manifested marked immunoreactivity for pAKT (S2F and S2K Fig), pGSK-3β (S2G and S2L Fig), and PCNA (S2I and S2N Fig). Immunoreactivity for CD31 was not detected in the tumor tissue (S2H and S2M Fig).

### Phenotypes of *pten* dko embryos

**Fig 4:**
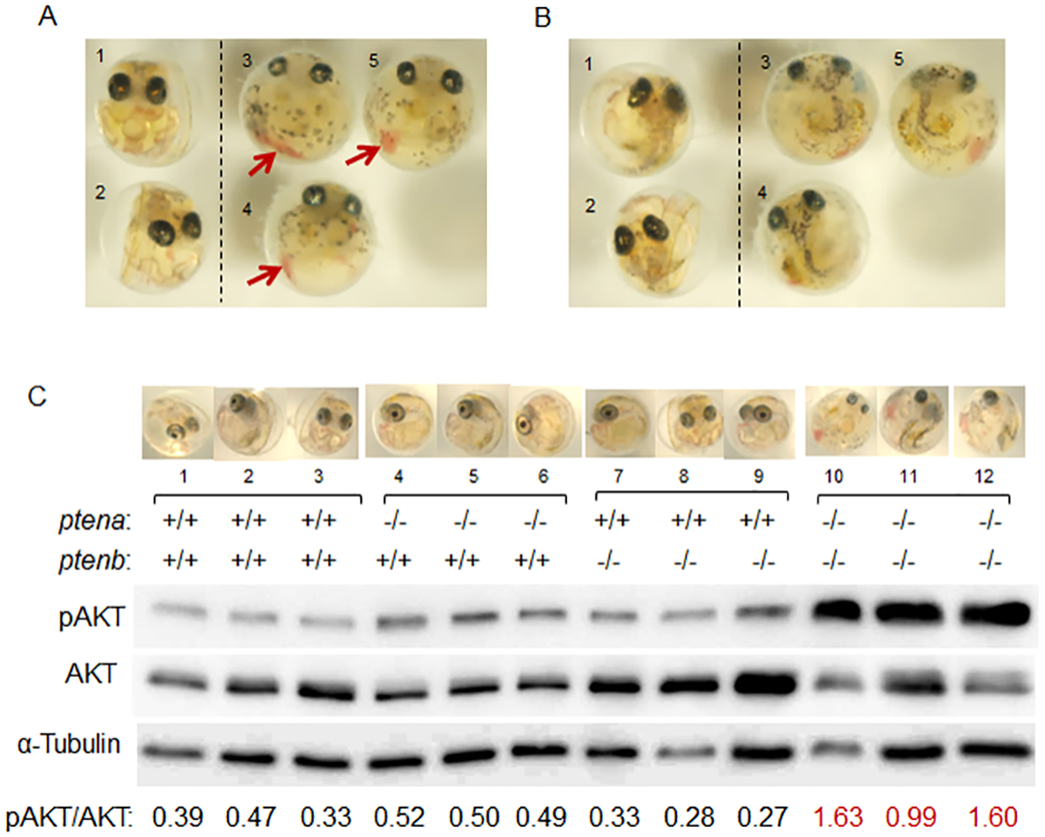
Phenotypes of and PI3K-AKT signaling in *ptena*^−/−^ *ptenb*^−/−^ medaka embryos. **(A**, **B**) Front (A) and back (B) views of the same *pten* dko medaka and their littermates at 8 dpf. The genotypes are *ptena*^+/−^*ptenb*^−/−^ (1), *ptena*^+/+^*ptenb*^−/−^ (2), and *ptena*^−/−^*ptenb*^−/−^ (3–5). The eyes of the dko embryos are smaller than those of their littermates, their vasculature is undeveloped with stagnated blood cells being evident (arrows), and their tails are short and curved. (**C**) Extracts (7.5 μg of protein) prepared from individual medaka embryos of the indicated *pten* genotypes at 6 dpf were subjected to immunoblot analysis for determination of the pAKT/AKT ratio. Each lane corresponds to an individual. The average pAKT/AKT ratio in dko embryos was about four times that in wild-type embryos (1.41 versus 0.40). Similar results were obtained in other two independent experiments. Given that the embryos shown in (C) were obtained in different experiment from embryos in (A, B), the numbers labeling individual embryos in A and B do not correspond to the numbers shown in C.

Although the theoretical segregation ratio for F1 *ptena*^−/−^*ptenb*^−/−^ progeny of *ptena*^+/−^*ptenb*^−/−^ crosses is 0.25, we did not detect any fish with the double-negative genotype at 2 mpf. We did detect 35 *ptena*^−/−^*ptenb*^−/−^ embryos (30.4%) out of a total of 115 embryos, however. These *pten* dko embryos manifested severe abnormalities—including small eyes, failure of Cuvierian duct development, and a short curved tail—at 3 days post fertilization (dpf) (Fig 4A and 4B and S3 Fig). It was difficult to distinguish dko embryos from wild-type embryos at 48 h post fertilization (hpf). At 56 hpf, some embryos manifested “blood pools” that became enlarged in dko embryos but regressed in non-dko embryos. Cuvierian ducts are apparent in wild-type embryos at 72 hpf, but they were not observed in dko embryos at this time. The tip of the tail detaches from the yolk in wild-type embryos before 72 hpf, but it failed to do so in dko embryos until the end of their life. Wild-type embryos move actively within the egg envelope after 72 hpf, but the dko embryos did not. The diameter of the eyeball was measured for medaka with *ptena*^+/+^*ptenb*^+/+^, *ptena*^−/−^*ptenb*^+/+^, *ptena*^+/+^*ptenb*^−/−^, and *ptena*^−/−^*ptenb*^−/−^ genotypes at 6 to 7 dpf (S2 Table). The eyeball diameter was significantly smaller for the dko embryos than for embryos of each of the other three genotypes. The *pten* dko embryos died at 7 to 12 dpf without hatching. We thus did not observe any hatched young dko fish.

### PI3K-AKT signaling in *pten* mutant embryos

Immunoblot analysis of PI3K-AKT signaling in individual embryos of various *pten* genotypes revealed that the pAKT/AKT ratio was markedly greater in dko embryos than in wild-type, *ptena*^−/−^*ptenb*^+/+^, or *ptena*^+/+^*ptenb*^−/−^ embryos (Fig 4C). The differences in the pAKT/AKT ratio between the dko embryos and embryos of the other genotypes were statistically significant (*P* = 0.018, 0.024, and 0.016, respectively). We therefore considered that this ratio might be exploited as an indicator for effects of drug treatment.

### Effects of PI3K-AKT signaling inhibitors on *pten* dko embryos

**Fig 5:**
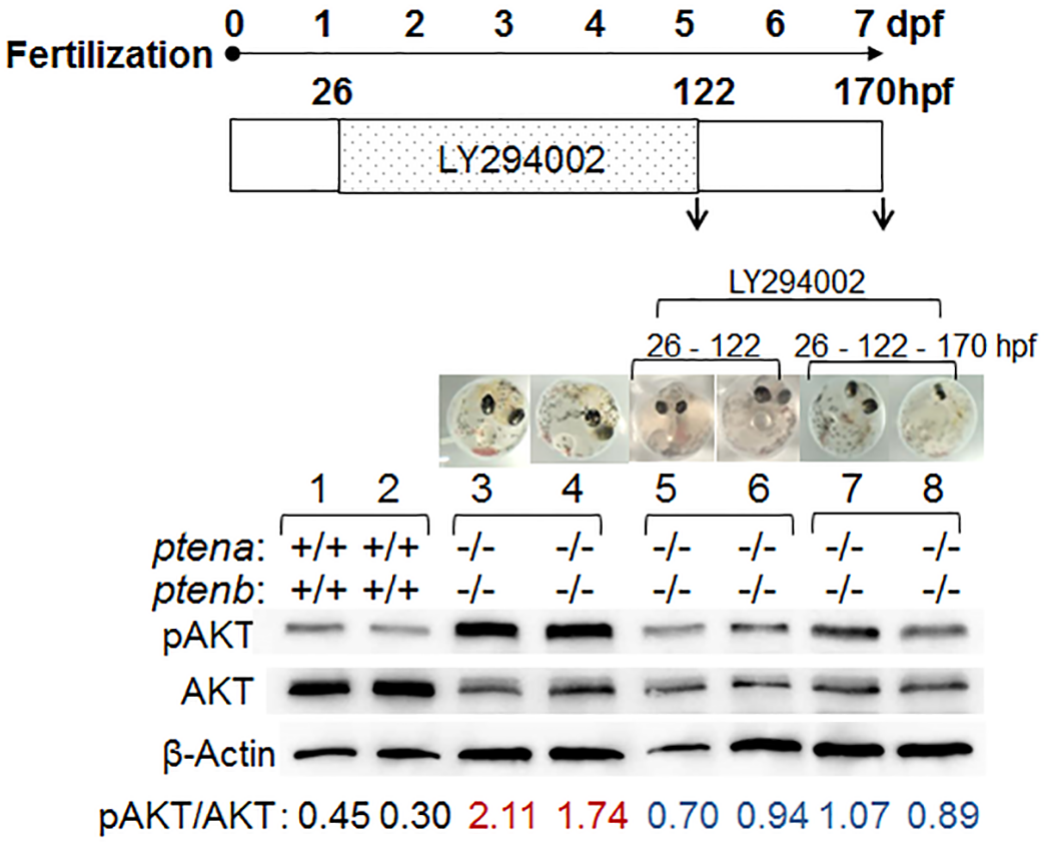
Effect of LY294002 on AKT phosphorylation in *pten* dko embryos. Wild-type or *pten* dko embryos were exposed (or not) to 15 μM LY294002 from 26 to 122 hpf and then either harvested or incubated in fish water without drug for an additional 48 h, as indicated. Extracts (5 μg of protein) prepared from individual embryos were then subjected to immunoblot analysis for determination of the pAKT/AKT ratio. Each lane corresponds to an individual. β-Actin was examined as a loading control. Similar results were obtained in other two independent experiments with similar conditions.

**Fig 6:**
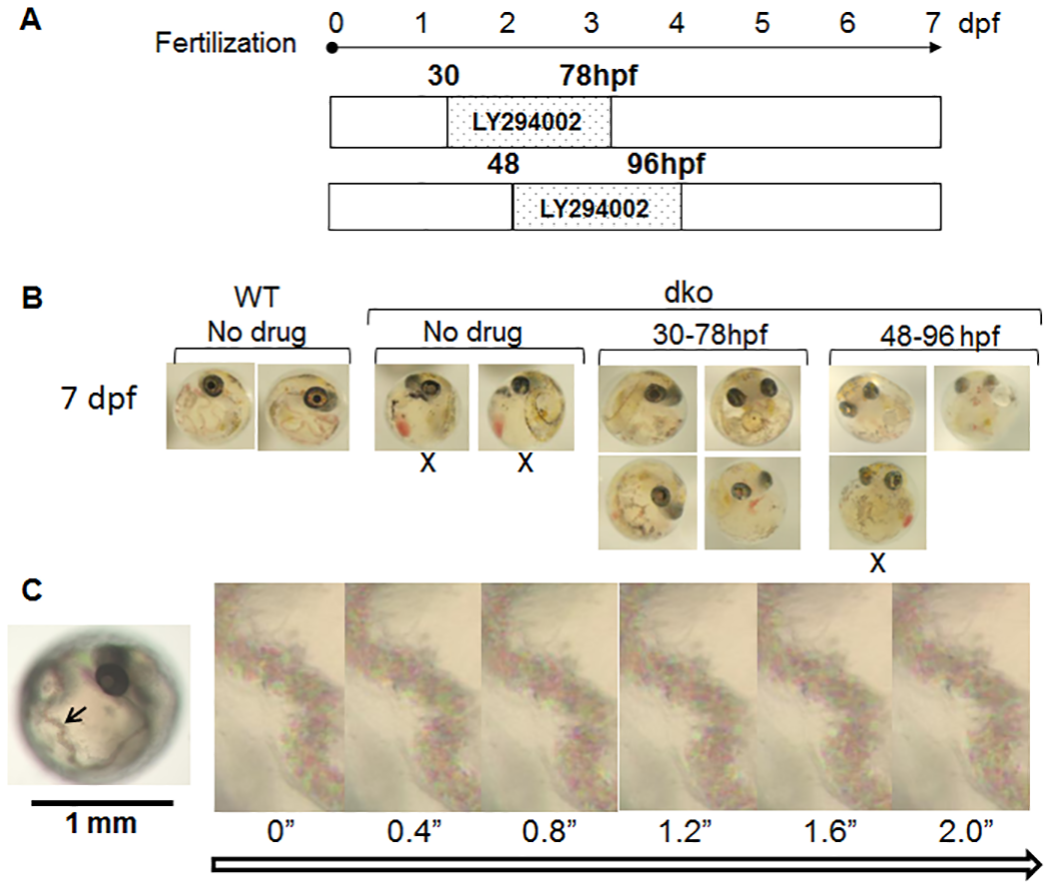
Effects of LY294002 on vascular development and blood cell flow in *pten* dko embryos. (**A**) Wild-type or *pten* dko embryos were exposed (or not) to 15 μM LY294002 for 48 h beginning at 30 or 48 hpf. (**B**) At 7 dpf, the embryos were photographed and genotyped. The dko embryos treated with LY294002 developed partial Cuvierian ducts (4 of 4 treated at 30 hpf, and 2 of 3 treated at 48 hpf). The dko embryos not exposed to the drug (2/2) did not manifest vasculogenesis. X, no duct. (**C**) Snapshots from a movie of a 4-dpf dko embryo that had been treated with LY294002 for 48 h beginning at 30 hpf. Blood cells can be seen flowing through the Cuvierian duct (arrow). Times are indicated in seconds.

We administered LY294002, a highly selective inhibitor of PI3K, to 26-hpf *pten* dko embryos for 96 h (Fig 5). The control experiment using wild-type embryos showed that there was no difference in the pAKT/AKT ratio among wild-type embryos with or without LY294002(S4 Fig). The pAKT/AKT ratio in the embryos treated with LY294002 was reduced to about one-half of that for nontreated embryos, indicating that a selective PI3K inhibitor was able to compensate for the loss of PTEN function in vivo and that this system is suitable for screening for drugs with such activity. We next examined whether LY294002-treated dko embryos were able to hatch (S5 Fig). Crossing of *ptena*^+/−^*ptenb*^−/−^ fish and treatment of the resulting embryos with LY294002 yielded one dko offspring out of 129 hatched young fish (S5A and S5B Fig and S6 Movie), indicating again that LY294002 is able to compensate for deficiency of PTEN function, albeit at a low efficiency with regard to restoration of hatching. To examine the effect of LY294002 on vascular development and blood flow, we administered the drug to dko embryos at 30 or 48 hpf for 48 h (Fig 6A). Most of the dko embryos (6/7) treated with the drug developed partial Cuvierian ducts (Fig 6B, S4 and S5 Movies), whereas those not treated did not (Fig 6B, S3 Movie). We also observed flow of blood cells through the Cuvierian duct of a 4-dpf dko embryo treated with LY294002 at 30 hpf (Fig 6C). There were large differences in phenotypes other than that of the Cuvierian duct among dko embryos treated with LY294002. Given that dko embryos have smaller eyes and shorter tails, these phenotypes may also serve as indicators for drug effects other than the PI3K-AKT pathway. Hatching is not suitable as such an indicator because of the low frequency of drug rescue in this regard.

**Fig 7:**
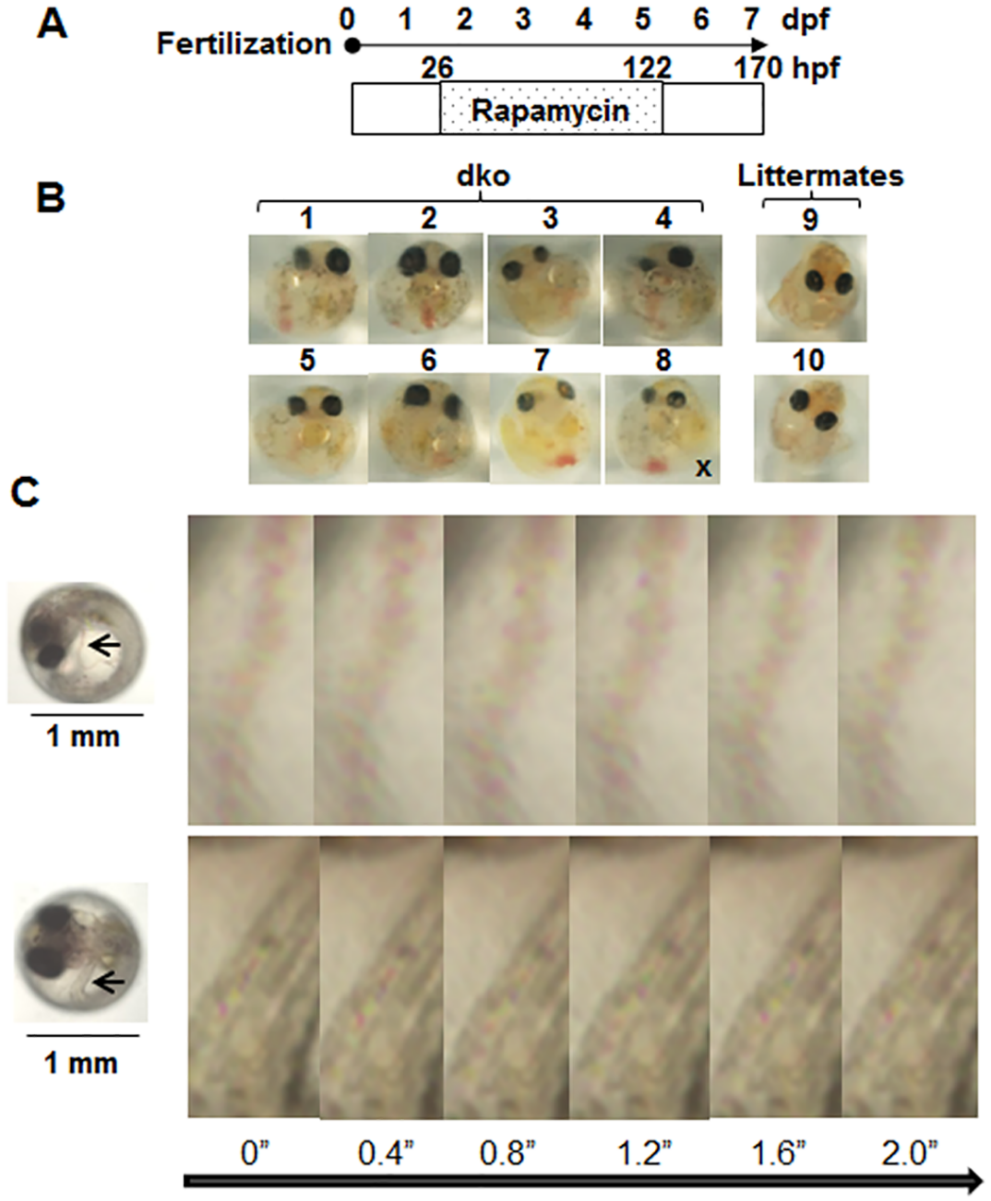
Effects of rapamycin on *pten* dko embryos. (**A**) A total of 41 embryos obtained from *ptena*^+/−^*ptenb*^−/−^ parents was treated with 5 μM rapamycin for 96 h beginning at 26 hpf. (**B**) At 7 dpf, the embryos were photographed and genotyped. Eight of the 41 embryos had the *ptena*^−/−^*ptenb*^−/−^ genotype (images 1–8), nine were *ptena*^+/−^*ptenb*^−/−^(image 9), and 10 were *ptena*^+/+^*ptenb*^−/−^(image 10). Partial Cuvierian ducts developed in seven of the eight dko embryos. X, no duct. (**C**) Snapshots from movies of the dko embryos shown in images 2 (upper) and 4 (lower) in (B). Blood cells can be seen flowing through the Cuvierian duct (upper) and the tail vascular duct (lower) indicated by the arrows. Times are in seconds.

We also tested the effects of other inhibitors of the PI3K-AKT pathway—including rapamycin, an inhibitor of mechanistic target of rapamycin (mTOR), and *N*-α-tosyl-L-phenylalanyl chloromethyl ketone (TPCK), an inhibitor of p70 ribosomal protein S6 kinase 1 (S6K1)—on *pten* dko embryos (Fig 7A, S6A Fig and S8–S10 Movies). The dko embryos treated with rapamycin or TPCK at 56 hpf manifested partial Cuvierian ducts (2 of 2 embryos treated with rapamycin, and 1 of 2 treated with TPCK) at 7 dpf (S6B Fig). We also observed flow of blood cells through the Cuvierian duct at 7 dpf in 7 of 8 dko embryos that had been treated with rapamycin at 26 hpf (Fig 7B and 7C). In addition, 3 (1, 2 and 6)out of 8 embryos(Fig 7B) dko embryos treated with rapamycin manifested the same or larger eyes compared with *ptena*^*+/−*^*ptenb*^*−/−*^(9) and *ptena*^*+/+*^*ptenb*^*−/−*^(10) littermates.

## Discussion

Our goal was to establish genetically engineered medaka fish as a system for screening of drugs able to compensate for loss of PTEN function. We have now shown that, similar to zebrafish, medaka possesses two functional *pten* genes. With the use of TALEN technology, we introduced mutations (deletions, insertions, and substitutions) into the exonic sequences of medaka *pten* genes. The sequences of the *ptena* #1 and *ptenb* #1 TALEN constructs were 82.4% identical (14/17 bp) in the left arm and 88.9% identical (16/18 bp) in the right arm. Despite this high sequence similarity, there was no instance in which a mutation occurred in *ptenb* with the *ptena* TALEN construct and vice versa, confirming the high specificity of TALEN technology. Homozygous single *pten* mutants of medaka were found to be viable and fertile, similar to the corresponding mutants of zebrafish. Whereas wild-type zebrafish embryos hatch at 2 to 3 dpf, wild-type medaka embryos hatch at 7 to 9 dpf. Given that formation of the vasculature begins at ~48 hpf in both zebrafish and medaka, up to which time *pten* dko medaka embryos appear to develop normally, this species difference in hatching time likely explains why *pten* dko medaka do not hatch whereas *pten* dko zebrafish do. All *pten* dko medaka embryos thus died between 7 and 12 dpf without hatching.

The phenotypes of *pten* dko medaka and zebrafish differ substantially with regard to vasculogenesis. Whereas dko embryos of zebrafish manifest a hyperbranching vasculature in the tail [13] [14], we found that the Cuvierian duct failed to develop in dko medaka embryos. In wild-type medaka embryos, blood is pumped from the heart at ~50 hpf[18], with obvious blood vessels being visible at 72 hpf under a stereomicroscope at low magnification (S3 Fig). In dko medaka embryos, however, we did not detect the Cuvierian duct on the yolk sac. Failure of duct development and pooling of blood cells is a severe phenotype, and we were thus able to identify *ptena*^−/−^*ptenb*^−/−^ embryos without genotyping as early as 72 hpf. This early identification of the mutant embryos is advantageous in that tumor formation is a relatively late event in the lifetimes of most rodent or fish models of tumorigenesis, necessitating animal maintenance for long periods of time (6 months or longer) as well as substantial amounts of test drugs. In our model, results of drug screening are obtained within 3 to 4 days and only small amounts of drugs are required.

Administration of the PI3K inhibitor LY294002 partially rescued the vascularization phenotype of *pten* dko medaka embryos so that we were able to detect blood flow through the partial Cuvierian ducts. Other inhibitors of the PI3K-AKT signaling pathway, including the mTOR inhibitor rapamycin and the S6K1 inhibitor TPCK, also rescued this mutant phenotype. These results suggest that the PI3K-AKT pathway is required for formation of the Cuvierian duct, and that this mutant phenotype is a suitable indicator in screening for drugs that target this pathway.

Immunoblot analysis showed that the pAKT/AKT ratio in *pten* dko medaka embryos was about four times that in wild-type embryos and that this increase was attenuated by treatment of the mutant embryos with LY294002. This compensation by drug treatment for deficiency of PTEN function can also serve as an indicator for the screening of drugs that act upstream of AKT in the PI3K-AKT signaling pathway.

We did not detect ocular tumors in adult *ptenb*^−/−^ medaka, whereas such tumors were apparent in adult *ptenb*^−/−^ zebrafish [19]. We did observe tumors on the trunk of *ptena*^+/−^*ptenb*^−/−^ adult medaka. It is not likely that these tumors are hemangiosarcoma, given that we did not detect CD31 immunoreactivity associated with them.

The eyeballs of *pten* dko medaka embryos were significantly smaller than those of embryos with other *pten* genotypes. The dko/wild-type ratio for average eyeball diameter was 0.22/0.31 mm, or 0.71. This mutant phenotype would be diagnosed as microphthalmia in humans (ratio of <0.84; anteroposterior diameter of the globe of <20 mm compared with the mean maximum axial length of the adult human eye of 23.8 mm) [20]. As far as we are aware, an association of *PTEN* mutation with microphthalmia in humans and mice has not been reported.

With regard to drug screening with *pten* dko medaka, we could not identify the dko embryos by their appearance at 48 hpf. The blood-pooling phenotype is apparent in some dko embryos at 52 hpf but, as a result of their slow growth, it is not apparent in others until after 72 hpf. We were able to identify dko embryos without genotyping at 72 hpf. Drug treatment of embryos derived from *ptena*^+/−^*ptenb*^−/−^ parents beginning before 52 hpf necessitates the use of embryos with all genotypes, whereas treatment starting at or after 72 hpf can be performed with identified dko mutants, but in this instance the drug effects are less pronounced. The optimal initiation point for drug treatment thus needs to be determined in future studies.

The vascularization impairment in *pten* dko medaka was partially but efficiently rescued by PI3K-AKT signaling inhibitors, whereas the hatching, eyeball size, and tail detachment defects were less efficiently rescued and may be attributable to loss of functions of PTEN other than regulation of PI3K-AKT signaling. These latter phenotypes may also serve as indicators in screening for drugs that compensate for PTEN deficiency, the identification of which should then provide further insight into PTEN function. In conclusion, we are able to distinguish *pten* dko medaka embryos from wild-type embryos without genotyping at 3 dpf. Our model system is thus likely to allow rapid screening for drugs able to compensate for PTEN deficiency with the use of only small drug amounts.

## Supporting information

S1 Fig Phenotypes of *ptena* or *ptenb* heterozygous or homozygous male fish at 7 mpf. Wild-type fish (**A**) and mutants manifesting muscle hyperplasia (**B**), a thin body (**C**, **F**, **G**), or abnormal osteogenesis (**D**, **E**) are shown. *ptenb* fish have no obvious phenotype(H, I). The numbers after *ptena* or *ptenb* correspond to the strains shown in Fig 2.
(PDF)

S2 Fig Phenotypes and histology of *ptena*^+/−^*ptenb*^−/−^ fish. (**A**) A fish with a tumor (arrow) at 8 mpf. (**B**) A fish with tumors (arrows) at 7 mpf. (**C**) A fish with a tumor (arrow) at 4 mpf. (**D**) A fish with abnormal osteogenesis at 4 mpf. (**E**–**N**) The tumor region in (A) was analyzed by hematoxylin-eosin (HE) staining (E, J) as well as by immunohistochemical staining for pAKT (F, K), pGSK-3β (G, L), CD31 (H, M), and PCNA (I, N). Images in the upper panels are shown at higher magnification in the lower panels. A scale bar under the upper panels indicates 500 μm. A scale bar under the lower panels indicates 100 μm.
(PDF)

S3 Fig Development of *pten* dko medaka embryos. At 48 hpf, blood flow was apparent from the heart in both wild-type and *pten* dko embryos. At 56 hpf, some dko embryos manifest blood pools near the tip of the tail that become larger with further development. At 72 hpf, wild-type embryos have an obvious Cuvierian duct (cd) whereas the dko embryos do not. The tail detaches from the yolk in wild-type embryos before 72 hpf, but it remains adhered to the yolk in dko embryos (arrows at 6 dpf). In wild-type embryos, nutrients in the yolk are absorbed and the size of the yolk sac gradually diminishes. In dko embryos, neither a Cuvierian duct on the yolk nor the vein in the tail are apparent, the size of the yolk sac does not decrease, and the tail is underdeveloped.
(PDF)

S4 Fig Effect of LY294002 on wild-type embryos. (**A**) Embryos were exposed to 15 μM LY294002 for 48 or 96 h beginning at 26 hpf. (**B**) At 7 dpf, extracts (5 μg of protein) were prepared from individual embryos and were subjected to immunoblot analysis for determination of the pAKT/AKT ratio. Each lane corresponds to an individual. β-Actin was examined as a loading control. Similar results were obtained in other independent experiment with same conditions.
(PDF)

S5 Fig Effect of LY294002 on hatching of *pten* dko embryos. **(A**) Crossing of *ptena*^+/−^*ptenb*^−/−^parents yielded 216 embryos that were exposed to 15 μM LY294002 for 48 or 96 h beginning at 26 or 48 hpf. Of the total of 216 embryos, 129 hatched and 87 died between 6 and 14 dpf without hatching. All embryos had the *ptenb*^−/−^genotype. (**B**) One embryo with the *pten* dko genotype hatched and developed a Cuvierian duct, but it could not swim and had a short curved tail.
(PDF)

S6 Fig Effects of rapamycin and TPCK on *pten* dko embryos. (**A**) Embryos were exposed to 5 μM rapamycin or 2 μM TPCK for 90 h beginning at 56 hpf. (**B**) At 7 dpf, the embryos were photographed and genotyped. The dko embryos treated with rapamycin (two of two) or TPCK (one of two) developed partial Cuvierian ducts, whereas those not exposed to drug did not manifest vasculogenesis. X, no duct. (**C**) Snapshots (0.4-s intervals) from a movie of the dko embryo shown in image 4 in (B). Blood cells can be seen flowing through the Cuvierian duct (arrow).
(PDF)

S1 Table Genotypes of progeny (2–3 mpf young or adult fish) generated by crossing *ptena*^−/−^*ptenb*^*+/−*^ and *ptena*^+/−^*ptenb*^−/−^ fish. (PDF)

S2 Table Diameter of the eyeball for medaka embryos of the indicated *pten* genotypes at 6 to 7 dpf as measured from enlarged photographic images. (PDF)

S1 Movie Wild-type embryo not treated with drug. (MP4)

S2 Movie Magnified view of the tail of the wild-type embryo in S1. (MP4)

S3 Movie A *pten* dko embryo not treated with drug. (MP4)

S4 Movie A dko embryo treated with 15 μM LY294002 for 48 h beginning at 30 hpf. (MP4)

S5 Movie A dko embryo treated with 15 μM LY294002 for 48 h beginning at 48 hpf. (MP4)

S6 Movie A dko embryo treated with 15 μM LY294002 for 96 h beginning at 26 hpf that hatched and developed a Cuvierian duct (arrow). (MP4)

S7 Movie One of four dko embryos treated with 15 μM LY294002 for 96 h beginning at 72 hpf developed partial blood vessels in which blood flow was detected. (MP4)

S8 Movie A dko embryo treated with 2 μM TPCK for 90 h beginning at 56 hpf. It did not develop a Cuvierian duct but its heart did begin to beat.
(MP4)

S9 Movie A dko embryo treated with 2 μM TPCK for 90 h beginning at 56 hpf. It manifested duct development and blood flow.
(MP4)

S10 Movie A dko embryo treated with 5 μM rapamycin for 96 h beginning at 26 hpf. The embryo developed a Cuvierian duct and blood cell circulation.
(MP4)

S1 Arrive Checklist (PDF)
