# Supplementary material for: Establishment of pten knockout medaka with transcription activator–like effector nucleases (TALENs) as a model of PTEN deficiency disease
Source: PLoS One. 2017 Oct 20;12(10):e0186878. doi: 10.1371/journal.pone.0186878 (PMC5650176; doi:10.1371/journal.pone.0186878)
Supplement: S1 Fig — Wild-type fish (A) and mutants manifesting muscle hyperplasia (B), a thin body (C, F, G), or abnormal osteogenesis (D, E) are shown. ptenb fish have no obvious phenotype(H, I). The numbers after ptena or ptenb correspond to the strains shown in Fig 2. (PDF) [file pone.0186878.s001.pdf]

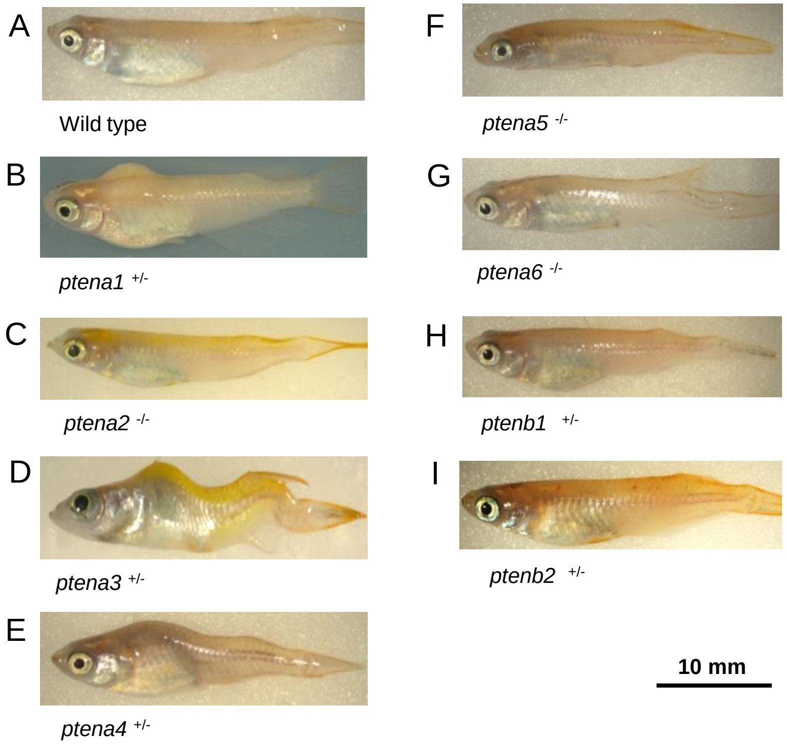

A
Wild type
B
ptena1 +/-
C
ptena2 -/-
D
ptena3 +/-
E
ptena4 +/-
F
ptena5 -/-
G
ptena6 -/-
H
ptenb1 +/-
I
ptenb2 +/-
10 mm
