# Supplementary figures and images for: Establishment of pten knockout medaka with transcription activator–like effector nucleases (TALENs) as a model of PTEN deficiency disease
Source: PLoS One. 2017 Oct 20;12(10):e0186878. doi: 10.1371/journal.pone.0186878 (PMC5650176; doi:10.1371/journal.pone.0186878)

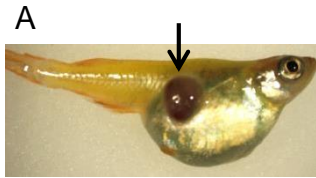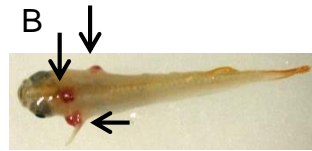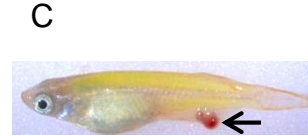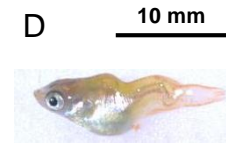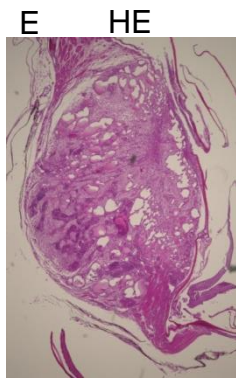

— : 500  $\mu$ m

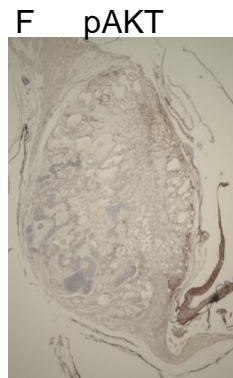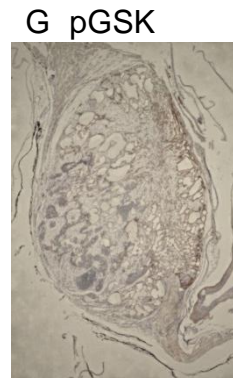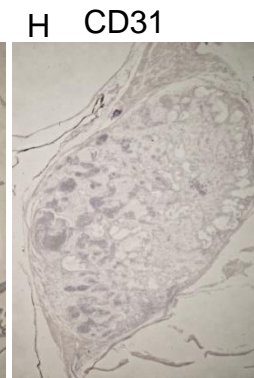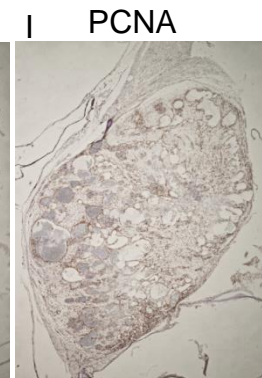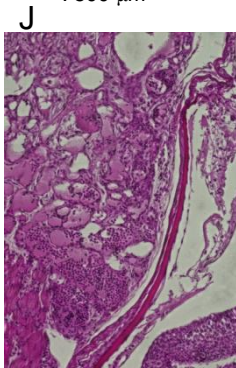

— : 100  $\mu$ m

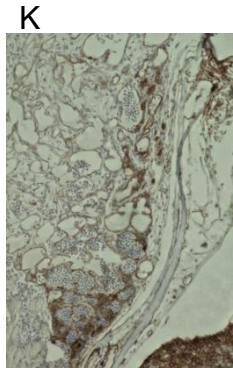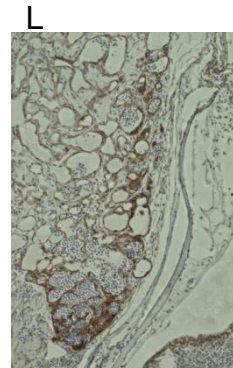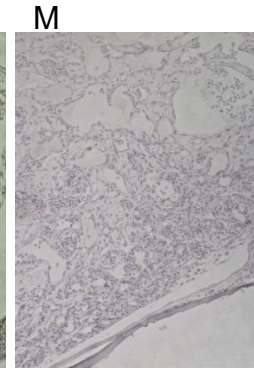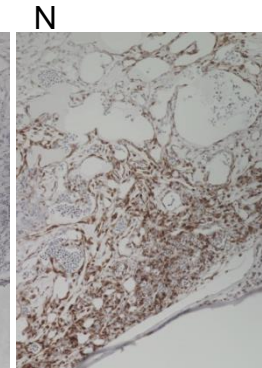

Supplement: S2 Fig — (A) A fish with a tumor (arrow) at 8 mpf. (B) A fish with tumors (arrows) at 7 mpf. (C) A fish with a tumor (arrow) at 4 mpf. (D) A fish with abnormal osteogenesis at 4 mpf. (E–N) The tumor region in (A) was analyzed by hematoxylin-eosin (HE) staining (E, J) as well as by immunohistochemical staining for pAKT (F, K), pGSK-3β (G, L), CD31 (H, M), and PCNA (I, N). Images in the upper panels are shown at higher magnification in the lower panels. A scale bar under the upper panels indicates 500 μm. A scale bar under the lower panels indicates 100 μm. (PDF) [file pone.0186878.s002.pdf]

48 hpf

56 hpf

72 hpf

6 dpf

Tip of the tail at 6 dpf

Wild-  
type

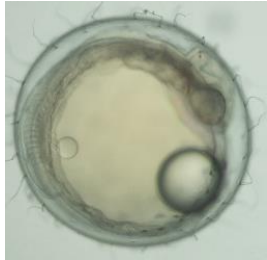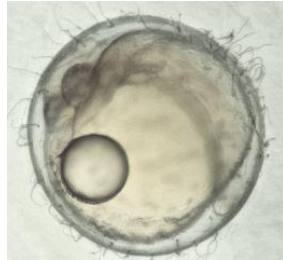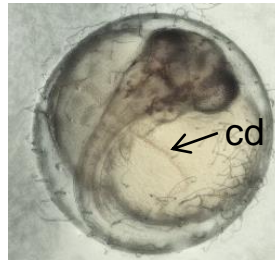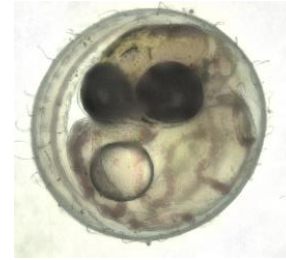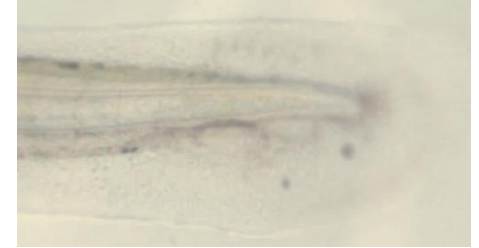

dko1

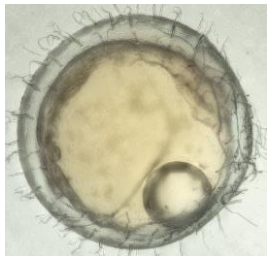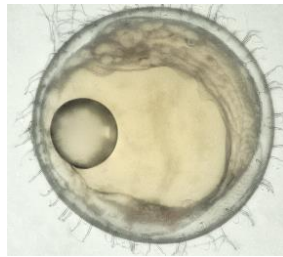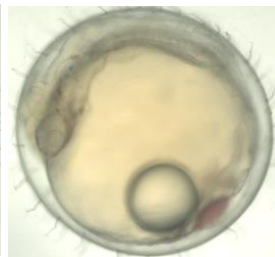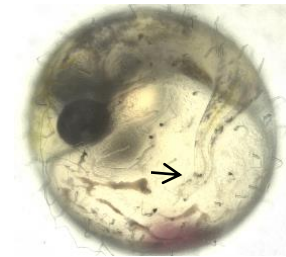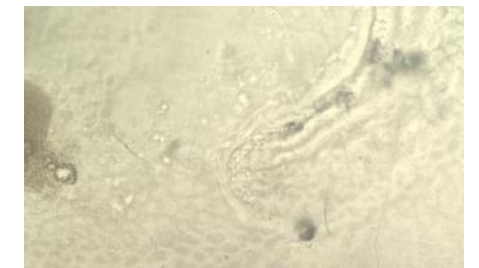

dko2

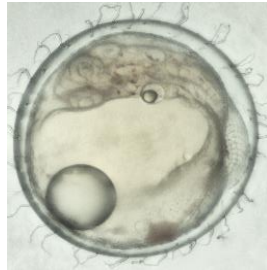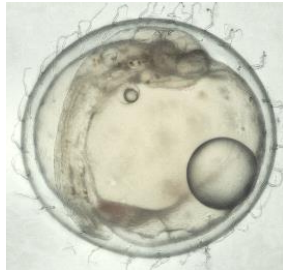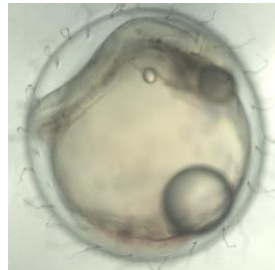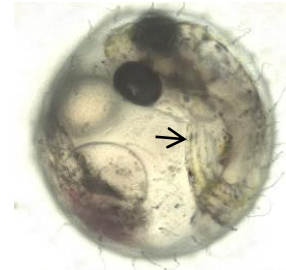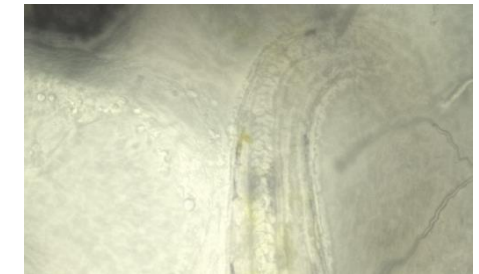

1 mm

Supplement: S3 Fig — At 48 hpf, blood flow was apparent from the heart in both wild-type and pten dko embryos. At 56 hpf, some dko embryos manifest blood pools near the tip of the tail that become larger with further development. At 72 hpf, wild-type embryos have an obvious Cuvierian duct (cd) whereas the dko embryos do not. The tail detaches from the yolk in wild-type embryos before 72 hpf, but it remains adhered to the yolk in dko embryos (arrows at 6 dpf). In wild-type embryos, nutrients in the yolk are absorbed and the size of the yolk sac gradually diminishes. In dko embryos, neither a Cuvierian duct on the yolk nor the vein in the tail are apparent, the size of the yolk sac does not decrease, and the tail is underdeveloped. (PDF) [file pone.0186878.s003.pdf]

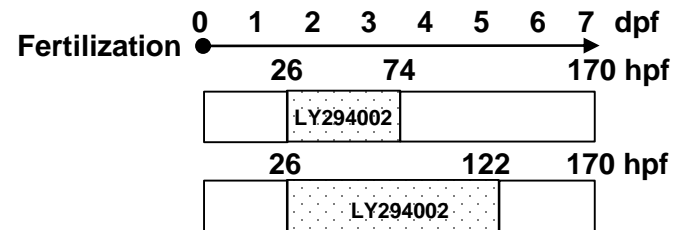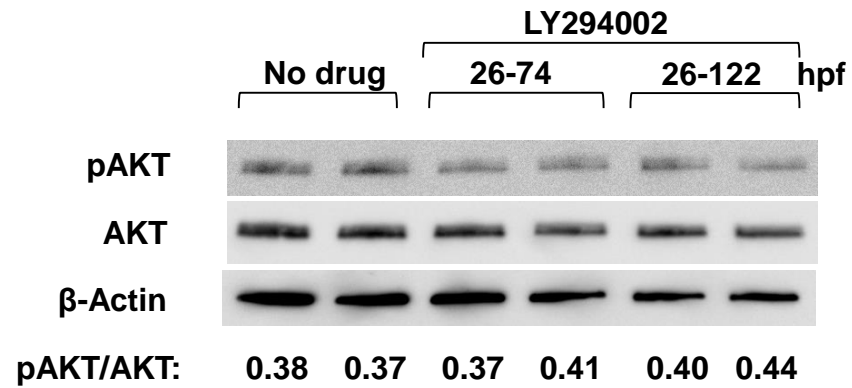

Supplement: S4 Fig — (A) Embryos were exposed to 15 μM LY294002 for 48 or 96 h beginning at 26 hpf. (B) At 7 dpf, extracts (5 μg of protein) were prepared from individual embryos and were subjected to immunoblot analysis for determination of the pAKT/AKT ratio. Each lane corresponds to an individual. β-Actin was examined as a loading control. Similar results were obtained in other independent experiment with same conditions. (PDF) [file pone.0186878.s004.pdf]

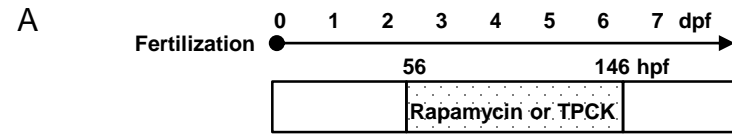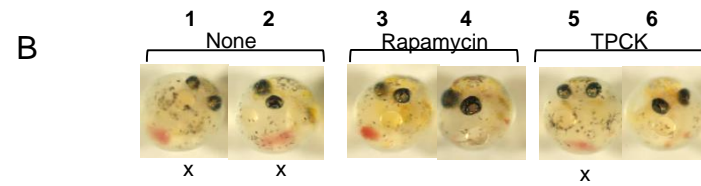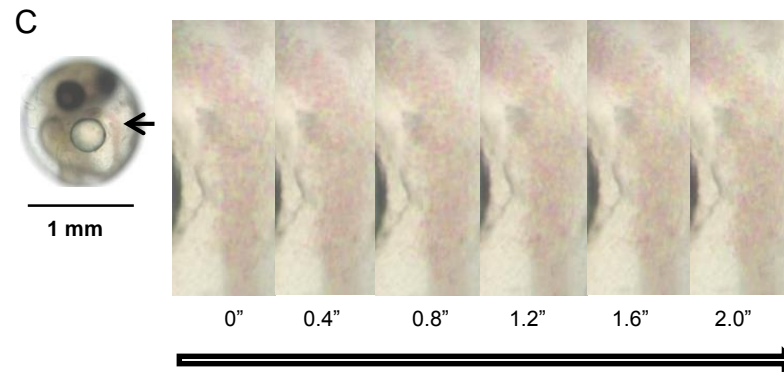

Supplement: S6 Fig — (A) Embryos were exposed to 5 μM rapamycin or 2 μM TPCK for 90 h beginning at 56 hpf. (B) At 7 dpf, the embryos were photographed and genotyped. The dko embryos treated with rapamycin (two of two) or TPCK (one of two) developed partial Cuvierian ducts, whereas those not exposed to drug did not manifest vasculogenesis. X, no duct. (C) Snapshots (0.4-s intervals) from a movie of the dko embryo shown in image 4 in (B). Blood cells can be seen flowing through the Cuvierian duct (arrow). (PDF) [file pone.0186878.s006.pdf]
