# Supplementary material for: Establishment of pten knockout medaka with transcription activator–like effector nucleases (TALENs) as a model of PTEN deficiency disease
Source: PLoS One. 2017 Oct 20;12(10):e0186878. doi: 10.1371/journal.pone.0186878 (PMC5650176; doi:10.1371/journal.pone.0186878)

A

			ptena genotype of hatched fish		
Embryos	Hatched	Hatching rate	+/+	+/-	-/-
216	129	0.60	35	93	1

B

Hatched embryos

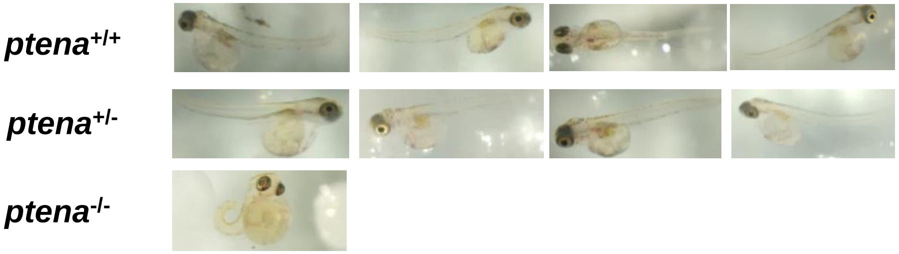

Supplement: S5 Fig — (A) Crossing of ptena+/−ptenb−/−parents yielded 216 embryos that were exposed to 15 μM LY294002 for 48 or 96 h beginning at 26 or 48 hpf. Of the total of 216 embryos, 129 hatched and 87 died between 6 and 14 dpf without hatching. All embryos had the ptenb−/−genotype. (B) One embryo with the pten dko genotype hatched and developed a Cuvierian duct, but it could not swim and had a short curved tail. (PDF) [file pone.0186878.s005.pdf]
