# Supplementary material for: Establishment of pten knockout medaka with transcription activator–like effector nucleases (TALENs) as a model of PTEN deficiency disease
Source: PLoS One. 2017 Oct 20;12(10):e0186878. doi: 10.1371/journal.pone.0186878 (PMC5650176; doi:10.1371/journal.pone.0186878)
Supplement: S1 Table — (PDF) [file pone.0186878.s007.pdf]

S1 Table. Genotypes of progeny (2-3 mpf young or adult fish) generated by crossing *ptena*$^{-/-}$*ptenb*$^{+/-}$ and *ptena*$^{+/-}$*ptenb*$^{-/-}$ fish

ptena	*ptenb*	Observed	Segregation	Theoretical
+/–	+/–	51	1	23.75
+/–	–/–	36	1	23.75
–/–	+/–	8	1	23.75
–/–	–/–	0	1	23.75

P values for the differences between observed and theoretical segregation ratios are as follows; *ptena* and *ptenb*, $*P = 1.78 \times 10^{-15}$; *ptena*, $*P = 5.26 \times 10^{-16}$; and *ptenb*, $*P = 0.018$.
