# Supplementary material for: Establishment of pten knockout medaka with transcription activator–like effector nucleases (TALENs) as a model of PTEN deficiency disease
Source: PLoS One. 2017 Oct 20;12(10):e0186878. doi: 10.1371/journal.pone.0186878 (PMC5650176; doi:10.1371/journal.pone.0186878)
Supplement: S2 Table — (PDF) [file pone.0186878.s008.pdf]

S2 Table. Diameter of the eyeball for medaka embryos of the indicated *pten* genotypes at 6 to 7 dpf as measured from enlarged photographic images

	ptena$^{+/+}$*ptenb*$^{+/+}$	*ptena*$^{-/-}$*ptenb*$^{+/+}$	*ptena*$^{+/+}$*ptenb*$^{-/-}$	*ptena*$^{-/-}$*ptenb*$^{-/-}$
n	24	20	30	20
Mean ± SD (mm)	0.31 ± 0.029[a]	0.29 ± 0.031[b]	0.30 ± 0.019[c]	0.22 ± 0.024

[a]$P = 7.04 \times 10^{-13}$, [b]$P = 5.91 \times 10^{-10}$, [c]$P = 2.43 \times 10^{-17}$ versus *ptena*$^{-/-}$*ptenb*$^{-/-}$.
